# Editorial Correspondence

**Published:** 1877-06

**Authors:** C. C. Hart

**Affiliations:** Cross Keys, Ga.


### EDITORIAL CORRESPONDENCE.

Cross Keys, Ga., May 15, 1877.

*Editors Atlanta Medical and Surgical Journal:*

I send you the following history of a case, which, to me, was an exceedingly interesting one during its progress.

On December 20th, 1876, I was called to see a colored boy, aged sixteen, and found him suffering excruciating pain in the umbilical region, with vomiting. I was informed that he had suffered a few days previously with diarrhœal discharges, which on the day before (15th) had suddenly ceased on carrying a heavy luggage to a neighboring house. His bowels had not been moved since the sudden arrest twenty-four hours before.

Finding no strangulated hernia to account for the symptoms, I adopted the usual treatment for painful cramp or spasm of the alimentary canal, without benefit. Opiates and enemas in large amount were used, without relief from pain or causing any fæcal discharge from the rectum.

Notwithstanding the frequent injection of large quantities of warm water into the colon, the stricture remained complete until the 25th, five days after treatment was commenced, when for the first time fæcal odor was detected. The discharges continued afterwards, and the patient gradually improved in every respect. On the 3d day of January, fourteen days after the attack, an obstruction was detected in the rectum, and with slight interference, a portion of the small intestine eighteen inches long was discharged. I have the specimen carefully preserved in alcohol, which I propose to exhibit as evidence of the curative power of nature.

The boy improved rapidly after the escape of the invaginated portion of bowel, and is now in good health.

On examination of the discharged portion of intestine, I find that it is a part of the small bowel, and on the internal or mucous surface a growth the size of a filbert, consisting of tough fribro-cartilaginous substance. This probably in some way led to the intussusception. Adhesion of the serous coat at the point of invagination doubtless prevented the escape of fæcal matter into the cavity of the peritoneum, after sloughing of the strangulated portion of bowel.

To me this is a remarkable case. Remarkable for the amount of intestine involved, and for the complete recovery by natural process. Strictures occur frequently, and are often attributed to intussusception, when there is no positive evidence of this condition. In this case, however, we have positive proof in the discharged portion of bowel.

C. C. Hart, M.D.

